# Constitutive Model Prediction and Flow Behavior Considering Strain Response in the Thermal Processing for the TA15 Titanium Alloy

**DOI:** 10.3390/ma11101985

**Published:** 2018-10-15

**Authors:** Jiang Li, Fuguo Li, Jun Cai

**Affiliations:** 1 State Key Laboratory of Solidification Processing, School of Materials Science and Engineering, Northwestern Polytechnical University, Xi’an 710072, China; 2 School of Metallurgical Engineering, Xi’an University of Architecture and Technology, Xi’an 710055, China; caijun0116@163.com

**Keywords:** TA15 titanium alloy, constitutive equation, strain compensation, flow stress

## Abstract

To investigate the flow stress, microstructure, and usability of TA15 titanium alloy, isothermal compression was tested at 1073–1223 K and strain rates of 10, 1, 0.1, 0.01, and 0.001 s^−1^, and strain of 0.9. The impact of strain and temperature on thermal deformation was investigated through the exponent-type Zener–Hollomon equation. Based on the influence of various material constants (including *α*, *n*, *Q*, and ln*A*) on the TA15 titanium alloy, the strain effect was included in the constitutive equation considering strain compensation, which is presented in this paper. The validity of the proposed constitutive equation was verified through the correlation coefficient (*R*) and the average absolute relative error (AARE), the values of which were 0.9929% and 6.85%, respectively. Research results demonstrated that the strain-based constitutive equation realizes consistency between the calculated flow stress and the measured stress of TA15 titanium alloy at high temperatures.

## 1. Introduction

TA15 alloy, a near-α titanium alloy, is extensively used in the aerospace industry due to its various advantages, such as weldability, mechanical capability at high temperatures, superior creep and erosion resistance, and large strength-to-weight ratio [1]. The mechanical properties and physical characteristics of TA15 titanium alloy have led to its wide application. The microstructure evolves in the process, and is influenced by process parameters: strain rate, strain, and temperature [2]. There is an interactive correlation between deformation behavior and microstructure evolution [3]. The required shape, properties, and microstructure can be obtained by optimizing the thermal deformation parameters in thermomechanical processing. Therefore, deformation and flow are being studied at elevated temperatures, which is conducive to investigating the thermal deformation ofTA15 titanium alloy.

Scholars have investigated the deformation and microstructural evolution of TA15 titanium alloy [4,5,6]. Processing the components is difficult given the complex shape, hard-to-deform properties, and requirement of forming quality [7]. Gao et al. studied the microstructure evolution and flow behavior of near-*α* titanium alloy, whose microstructure is inhomogeneous in thermal deformation, finding that nonuniform microstructure before deformation was composed of *β* phase, lamellar α, and equiaxed α in the colony form. There was a correlation between the Burgers orientation and *β* phase in α colony [6]. According to Zhao et al., thermal flow stress is not significantly influenced by the initial *β* grain size [5]. By investigating the influence of inhomogeneous deformation on the microstructure of the rib-web part of TA15 alloy, Fan et al. found four microstructures in the forming process [8]. The superplasticity of TA15 alloy was enhanced using thermomechanical techniques, which could refine the grains of the alloy [9]. Thermal uniaxial tensile tests were conducted and finite element models were established to explore the thermal formability of TA15 titanium alloy [10]. Zhu et al. explored the influence of cooling speed on the major *α* phase microstructure evolution of TA15 titanium alloy. According to the research results, the size distribution and volume fraction of the major α phase are influenced by cooling speed [11]. The kinetics rate and dynamic globularization kinetics of TA15 are influenced by deformation parameters. The dynamic globularized fraction is in direct proportion to strain and temperature, but inversely proportional to strain rate [12]. A prediction model was established on the basis of an improved back propagation (BP) neural network, and it was used to explore the quantitative evolution of aspect ratio, grain size, and volume fraction of equiaxed *α* for TA 15 alloy [13].

As a foundation of engineering parts production, thermal deformation processes require not only microstructural and mechanical properties, but also dimensional accuracy [14]. As excellent mechanical properties require fine microstructural characteristics, it was necessary to explore the thermomechanical process affecting the microstructural characteristics. To this end, we used theconstitutive equation at various temperatures and strain rates. The Arrhenius model, as a phenomenological constitutive model, has generally been used to present the correlations among temperature, flow stress, and strain rate in a constitutive study, particularly at high temperatures [15]. The influence of strain has been proven to be necessary to verify the constitutive equation involving the strain effect, which predicted flow behaviors at elevated temperatures in pure titanium [16] and titanium alloy [15], steel [17,18,19], aluminum alloy [14,20,21], and magnesium alloy [22].

This research attempted to represent thermal deformation of TA15 titanium alloy by formulating a proper constitutive correlation. The thermal compression was tested at different temperatures and strain rates. The flow stress was further analyzed based on the test results. A comprehensive constitutive model involving temperature, strain rate, and flow stress was established. Finally, the reliability of the constitutive model was verified.

## 2. Experimental Details

TA15 titanium alloy, a near-α titanium alloy that has the chemical composition of Ti-6.5Al-2Zr-1Mo-1V (in wt%), was used in the research. The original specimen was sectioned from the bar axial, ground to 2000 grit with sand paper, and then polished to 0.5 μm. Finally, the specimen was etched to carry out optical microscopy (GX51F, OLYMPUS, Tokyo, Japan) observation, and Kroll’s agent (2% HF, 4% HNO_3_, and 94% H_2_O) was used to etch the specimens for 3–5 s.

**Figure 1:**
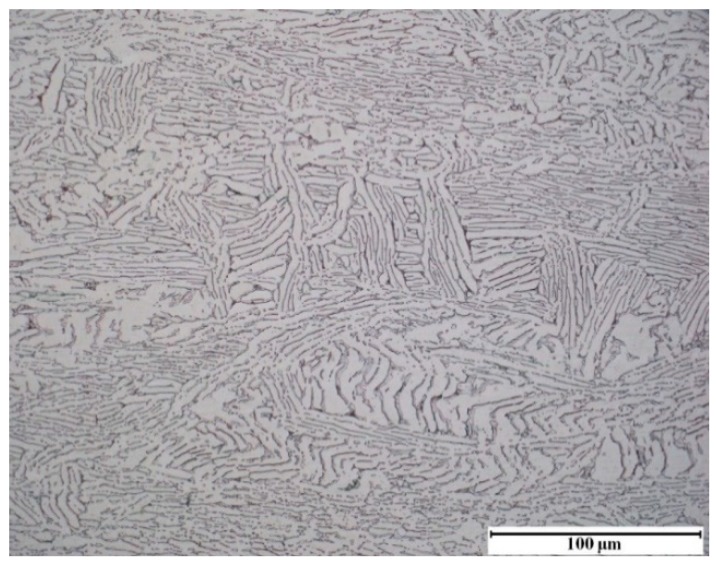
Initial microstructure of the specimens of TA15 titanium alloy.

The *β* transus temperature of TA15 was measured as approximately 1258 K, and all specimens used in the experiments were annealed for homogenization by heating at 1073 K for 1 h and cooling in the furnace. The microstructure shown in Figure 1 primarily consisted of a number of coarse strip-shaped *α* phases; meanwhile, there were only a few thin strip-shaped *α* phases on the *β* matrix.

**Figure 2:**
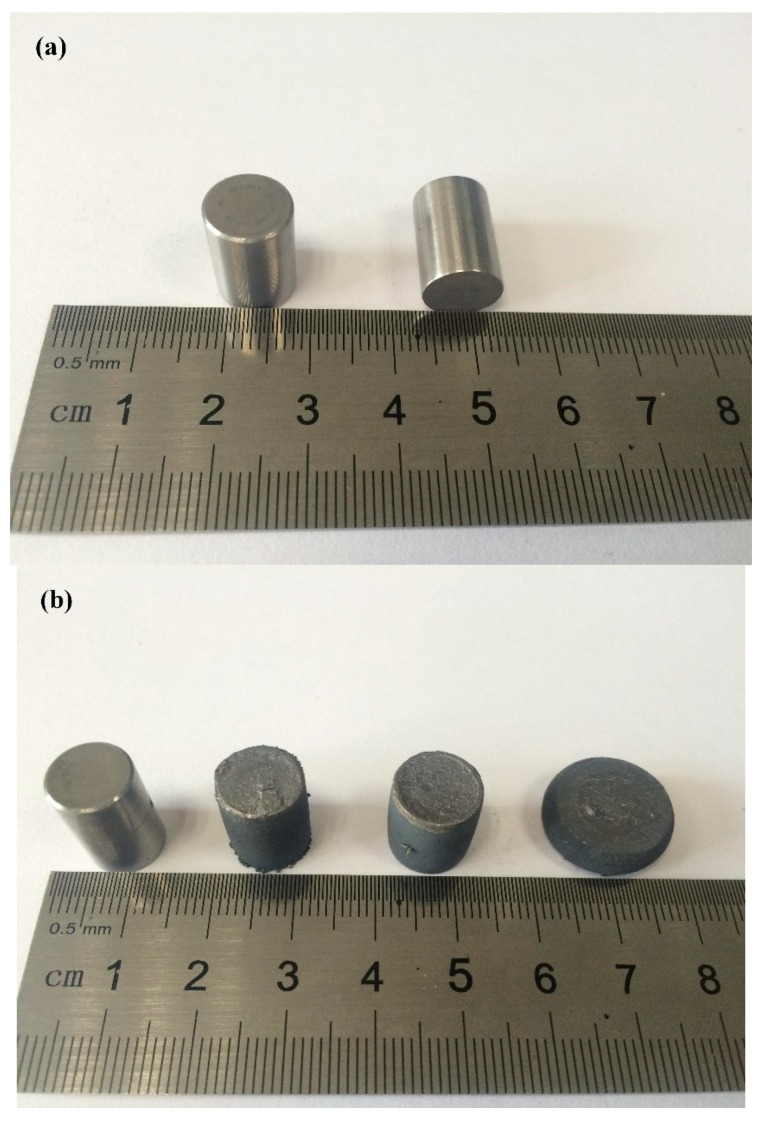
The appearance of the specimen: (**a**) before deformation and (**b**) after deformation.

The isothermal compression simulation test is the most commonly used method to investigate the deformation and microstructural evolution of materials at high temperatures. An isothermal compression simulation experiment was carried out on a Gleeble-3500 tester (DSI Corporation, New York, USA). Cylindrical *Ф*10 mm × 15 mm specimens were used in this study, as shown in Figure 2. The upper and lower end faces were parallel to each other. The mechanical perpendicularity of the vertical face was maintained, and the two end surfaces were smooth to decrease the effects of transverse friction on deformation. The axis of the cylindrical specimen was the axial line of the bar billet. In the experiment, a radial sensor was used in the deformation of the specimen to collect and record the cross-section area and the collected signals to control the parameters of the tester. The specimen was compressed at a constant strain rate. The whole test was electrically heated, and the temperature was obtained with a thermocouple. The temperature deviation was controlled in ±1 °C increments.

The isothermal simulation compression experiment temperature ranged from 1073 K to 1223 K in increments of 50 K. The strain rate varied from 0.001 s^−1^ to 10 s^−1^, and the corresponding reduction ranges were 10%, 20%, and 60%. A graphite flake cushion was used between the specimen and the pressure head to decrease transverse friction. We heated the specimen to deformation temperature at a heating rate of 10 °C/s, which was maintained for 3 min to guarantee temperature uniformity. Thermal deformation occurred at the isothermal constant strain rate. The stress-strain curves were automatically recorded as the isothermal compression at elevated temperature was tested. The specimens were quenched immediately in water after the compression test to maintain the organization.

## 3. Results and Discussion

### 3.1. Experiment Results and Flow Stress Behavior

**Figure 3:**
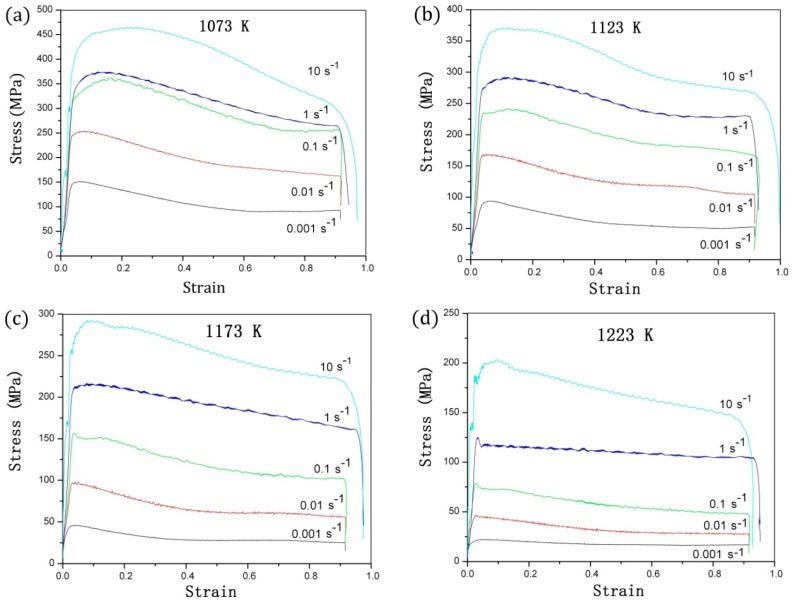
True stress versus true strain curves of TA15 titanium alloy obtained by isothermal hot compression tests at temperatures of: (**a**) 1073 K, (**b**) 1123 K, (**c**) 1173 K, and (**d**) 1223 K.

Figure 3 depicts the stress-strain curves of TA15 titanium alloy in various deformation conditions. The flow stress rises substantially as the strain increases due to the work-hardening resulting from the increasingly higher dislocation density. Flow stress showed peaks at nearly all temperatures and strain rates, and a clear flow softening phenomenon occurred. Peak strain was generally less than 0.1, and it did not change obviously with thermal deformation conditions.

As can be seen from Figure 3, the stress-strain curves of TA15 titanium alloy were obtained by testing the isothermal compression at 1073 K to 1223 K in intervals of 50 K. The flow stress first peaked and then decreased as the strain increased. In addition, flow stress was highly sensitive to temperature. It first sharply decreased and then flattened out as the temperature rose. We ignored the deformation heating due to the low strain rates (0.001 s^−1^ and 0.01 s^−1^). At 1173 K and 1223 K, the flow stress curves flattened out as the strain rate decreased. Formation at low strain rates can only enhance dynamic recovery (DRV), but can also inhibit dynamic recrystallization (DRX). Above the phase transition temperature, the *β* grain size was large as the temperature rose. The microstructure indicates that the DRV was dominant with little DRX during formation. At different experimental temperatures, the volume fraction and grain size of the major α phase were inversely proportional to temperature [4].

TA15 titanium alloy presented flow softening, which was closely related to the thermal processing conditions and the initial microstructure, which is common in the metal deformation process. The dynamic recrystallization was inhibited by DRV. The flow stress tended to be stable after saturation. A small amount of DRX occurred at higher strain. The *β*-transus temperature was 1263 K, and the DRV was the main softening mechanism in the *β*-phase field because of the body-centered cubic crystal structure with rapid self-diffusion and high stacking fault energy [23].

**Figure 4:**
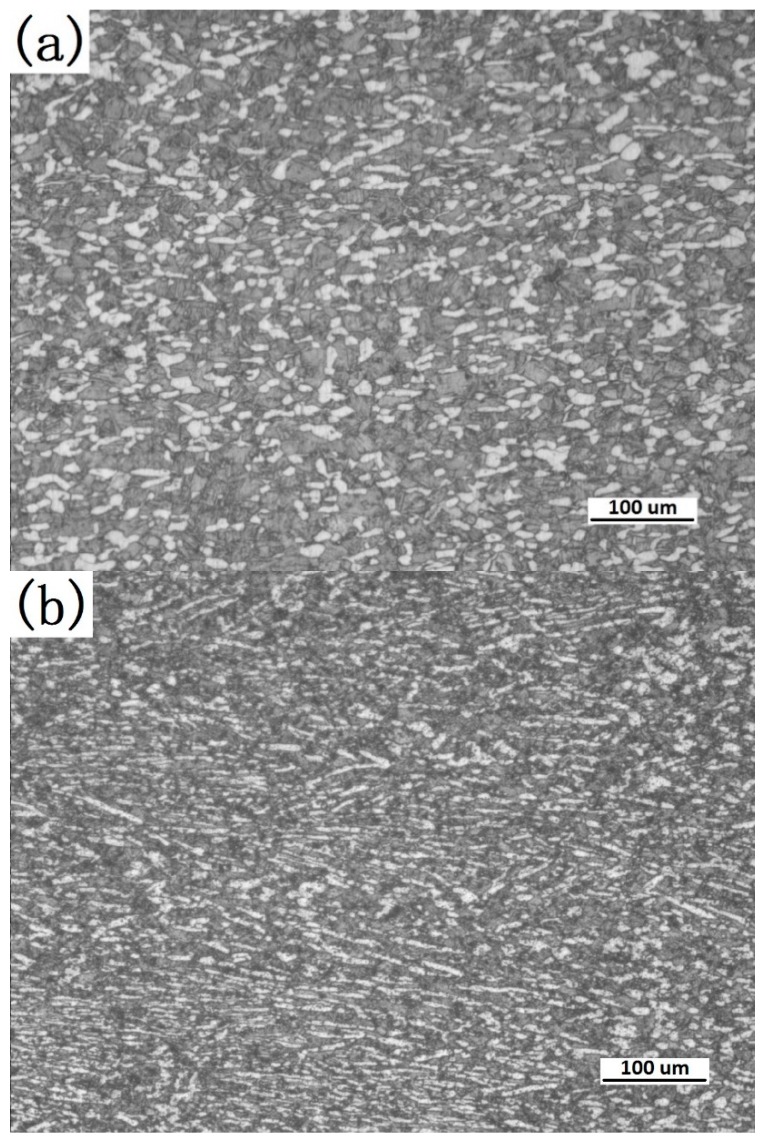

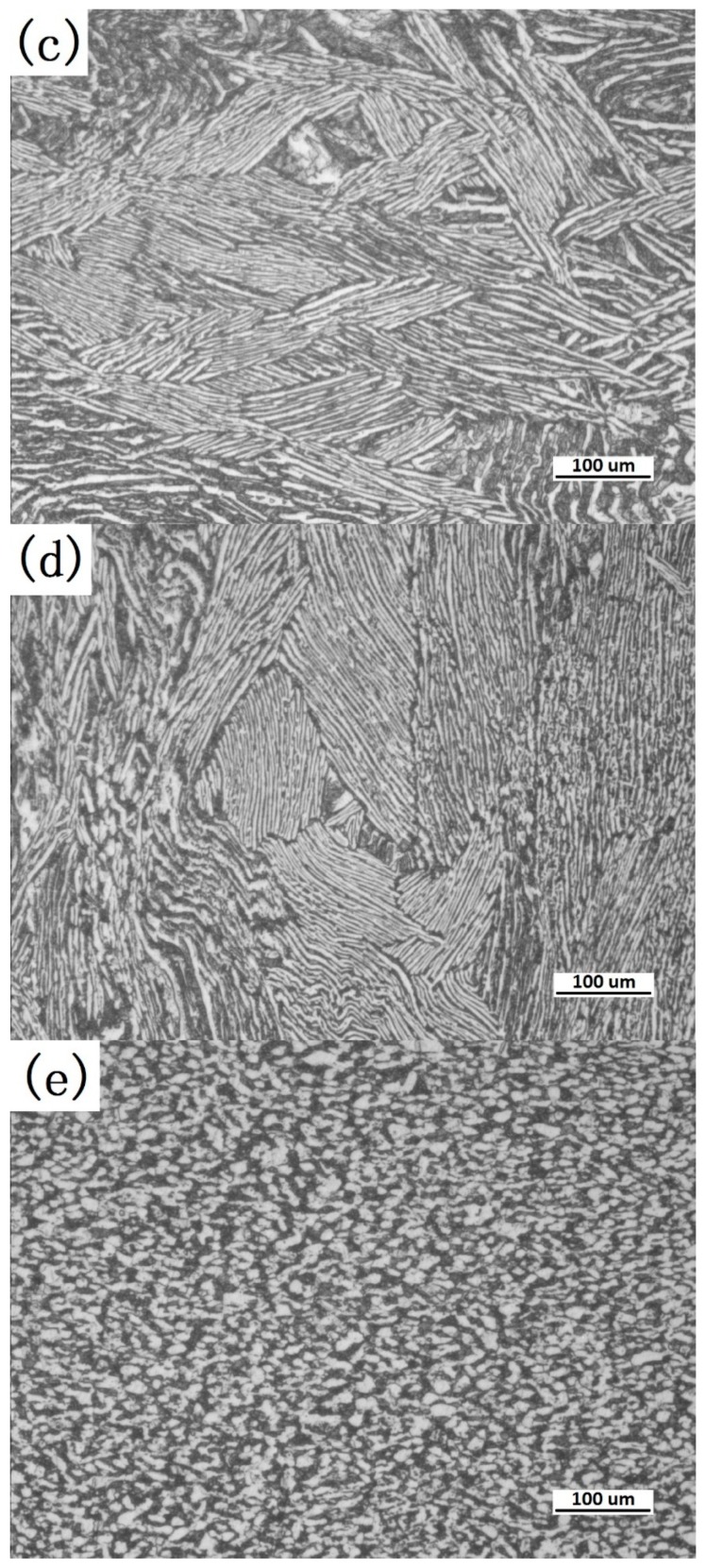
Microstructure of TA15 titanium alloy specimens compressed to strain 0.9 at 1223 K at different strain rates: (**a**) 0.001 s^−1^, (**b**) 0.01 s^−1^, (**c**) 0.1 s^−1^, (**d**) 1 s^−1^, and (**e**) 10 s^−1^.

The microstructure variations of the specimens at 1223 K are shown in Figure 4, and the deformation strain rates increased from 0.001 s^−1^ to 10 s^−1^. Figure 4a indicates that equiaxial recrystal grains were found, which replaced the initial and primary strip-shaped grain of 0.001 s^−1^, probably due to the deformation recrystallization of the microstructure under this deformation condition. As the deformation condition changed, the primary strip-shaped α phase volume fraction varied, and the *β* matrix volume fraction increased gradually with increasing deformation temperature. It can be seen from the change of the microstructure evolution that the recrystallization volume fraction was influenced by the strain rate and decreased as the latter increased. Considering the trend in the variation of the stress-strain curve under the corresponding conditions, the α phase and *β* phase recrystallizations were the potential reason for flow softening, as shown in Figure 4a–e. The flow softening mechanism is more complicated when deformed in the two-phase zone for titanium alloy. As shown in Figure 4c,d, the initial strip-shaped grain was elongated and smooth along the vertical compression direction, which proved that no obvious DRX occurred in this process. Some squashed β grains at 10 s^−1^ were observed and a few *β* matrix grains were compressed and partial *β* grains were equiaxial, which proved the occurrence of recrystallization in *β* phase, as shown in Figure 4e. The adiabatic shear band was another important reason for the flow softening, especially at the higher strain rate, such as 10 s^−1^ in this study; however, no obvious adiabatic shear band was found (Figure 4e).

### 3.2. Constitutive Modeling

#### 3.2.1. Constitutive Equation Derivation

The flow stress of TA15 titanium alloy was affected by the strain rate and temperature in the evaluated temperature deformation. The stress-strain curve was obtained by testing the isothermal compression at different temperatures and strain rates, so that the constants of the presented constitutive equation could be obtained.

The Arrhenius equation, which is a phenomenological model, was used to predict the constitutive equation [24], illustrate the correlation among temperature, flow stress and strain, and express the special parameter *Z* [25,26]:

(1) $$Z=\dot{\varepsilon }\exp\left(\frac{Q}{RT}\right)$$

(2) $$\dot{\varepsilon }=AF\left(\sigma \right)\exp\left(-\frac{Q}{RT}\right)$$

where

(3) $$F\left(\sigma \right)=\{\begin{array}{ll} \sigma^{n^{\prime }} & \alpha \sigma <0.8\\ \exp\left(\beta \sigma \right) & \alpha \sigma >1.2\\ {\left[\sinh\left(\alpha \sigma \right)\right]}^{n} & for\;all\;\sigma \end{array}$$

where *T* is absolute temperature in *K*, *R* is a gas constant, 8.3145 J/mol·K, *Q* is the deformation activation energy of thermal deformation in J/mol, *έ* is strain rate in s^−1^, and *A*, *n*’, *β*, *α*, and *n* are constants of materials, α = *β*/*n*’.

#### 3.2.2. Material Constants

Material constants in the constitutive equation were evaluated through the stress-strain curve based on experimental data. However, the strain effect was not considered in Equations (1) and (2). Previous research mainly studied the Arrhenius model, which accurately illustrates the correlation among flow stress, deformation temperature, and strain. The following process was evaluated at a strain of 0.2.

The values of *F*(*σ*) were replaced in Equation (2). Correlation between low stress and high stress is as follows:

(4) $$\dot{\varepsilon }=B\sigma^{n^{\prime }}\;(\mathrm{for}\;\alpha \sigma <0.8)$$

(5) $$\dot{\varepsilon }=C\exp\left(\beta \sigma \right)\;(\mathrm{for}\;\alpha \sigma >1.2)$$

where *B* and *C* are material constants that are independent from the deformation temperature. The natural logarithms of both sides of Equations (4) and (5) were taken, and the equations are as follows:

(6) $$\ln\left(\sigma \right)=\frac{1}{n^{\prime }}\ln\left(\dot{\varepsilon }\right)-\frac{1}{n^{\prime }}\ln\left(B\right)$$

(7) $$\sigma =\frac{1}{\beta }\ln\left(\dot{\varepsilon }\right)-\frac{1}{\beta }\ln\left(C\right)$$

**Figure 5:**
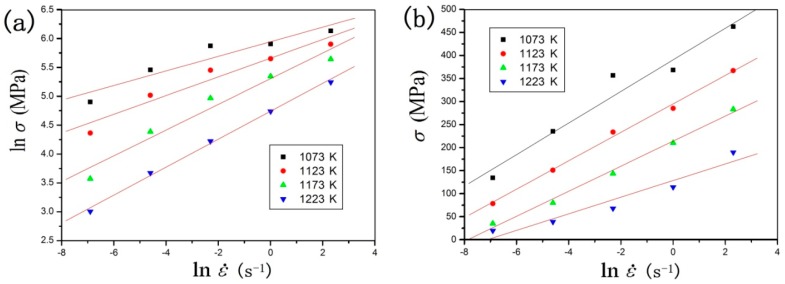
Evaluating the value of (**a**) *n*’ by plotting lnσ versus ln*έ* and (**b**) *β* by plotting *σ* versus ln*έ*.

The corresponding value under the strain of 0.2 was substituted into the above equations. The correlation plots of ln(*σ*)−ln(*έ*) and *σ*−ln(*έ*) were obviously approximated by parallel and straight lines, respectively. *β* and *n*’ were obtained from the line slopes in the ln(*σ*)−ln(*έ*) plot and *σ*−ln(*έ*) plot, as shown in the following equations and Figure 5. The averages of *n*’ and *β* were 5.6875 and 0.0384 MPa^−1^, respectively.

(8) $$n^{\prime }={\left[\frac{\partial \ln\dot{\varepsilon }}{\partial \ln\sigma }\right]}_{T}$$

(9) $$\beta ={\left[\frac{\partial \ln\dot{\varepsilon }}{\partial \sigma }\right]}_{T}$$

Subsequently, Equation (2) was rewritten for all strain levels as follows:

(10) $$\dot{\varepsilon }=A{\left[\sinh\left(\alpha \sigma \right)\right]}^{n}\exp\left(-\frac{Q}{RT}\right)$$

The natural logarithms of both sides were taken for the above equation. The equation is as follows:

(11) $$\ln\left[\sinh\left(\alpha \sigma \right)\right]=\frac{\ln\dot{\varepsilon }}{n}+\frac{Q}{nRT}-\frac{\ln A}{n}$$

**Figure 6:**
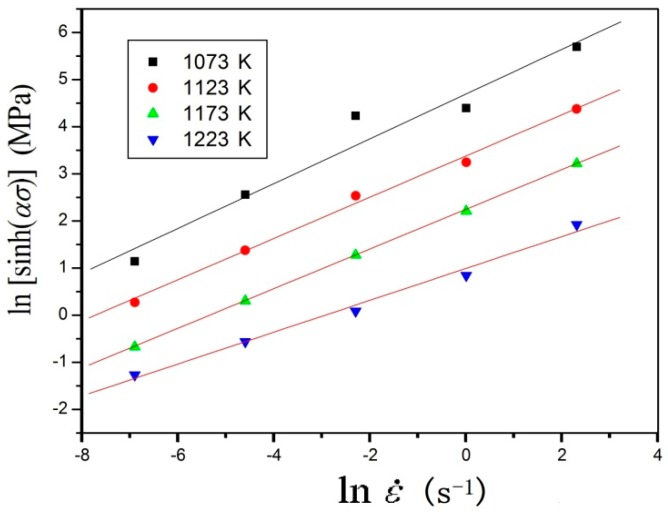
Evaluating the value of *n* by plotting ln[sinh(*ασ*)] versus ln*έ*.

At a certain temperature, after Equation (11) is differentiated:

(12) $$\frac{1}{n}={\left[\frac{\partial \ln\left[\sinh\left(\alpha \sigma \right)\right]}{\partial \ln\dot{\varepsilon }}\right]}_{T}$$

where the *n* value was taken based on the line slopes of ln[sinh(*ασ*)]−ln*έ* in Figure 6, which was 3.5987. And the value of ln*A* can be derived from the intercept of Figure 6.

At a specific strain rate, Equation (11) is differentiated:

(13) $$Q=R{\left[\frac{\partial \ln\dot{\varepsilon }}{\partial \ln\left[\sinh\left(\alpha \sigma \right)\right]}\right]}_{T}{\left[\frac{\partial \ln\left[\sinh\left(\alpha \sigma \right)\right]}{\partial \left(1/T\right)}\right]}_{\dot{\varepsilon }}$$

**Figure 7:**
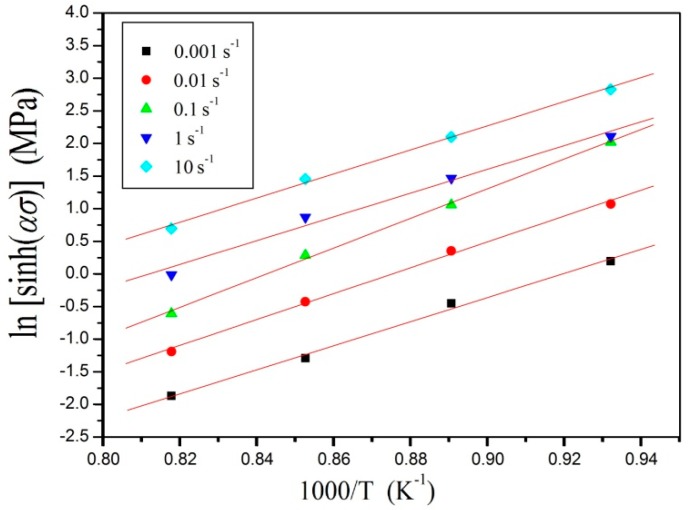
Evaluating the value of activation energy by plotting ln[sinh(*ασ*)] versus 1000/*T*.

As can be seen in Figure 7, when the strain was 0.2, flow stress and deformation temperatures at a certain strain rate were substituted to Equation (13), and the value of *Q* was derived in the plotting slope of ln[sinh(*ασ*)] as a function of 1/T (*Q* was in kJ/mol for ln[sinh(*ασ*)]−1000/*T*). Average *Q* at different strain rates was 584.63 kJ, and the value of *Q* was obtained, which was consistent with the current reference (654 kJ/mol by Zhang [27] and 588.7 kJ/mol by Luo [28] for *α* + *β* phase region). When deformation activation energy *Q* in deformation approached diffusion activation energy, the major material softening mechanism was dynamic recovery. When the diffusion activation energy was much lower than the deformation activation energy, the DRX may have been the main mechanism. According to the study of Briottet et al., the deformation activation energy of titanium alloy is high in the two-phase region due to the change in temperature, which changes the phase volume fraction and ultimately affects the flow stress through the modeling analysis [29]. The value of ln*A* at a certain strain was 57.1869, determined from the intercept of Figure 7. The dislocation climb, recrystallization, and recovery were related to *Q*, which reflected the characteristics of deformation [30].

Subsequently, similar processes were followed so that the above-mentioned material constants could be obtained at deformation strains ranging from 0.1 to 0.8 at 0.1 intervals.

#### 3.2.3. Strain Effect

**Figure 8:**
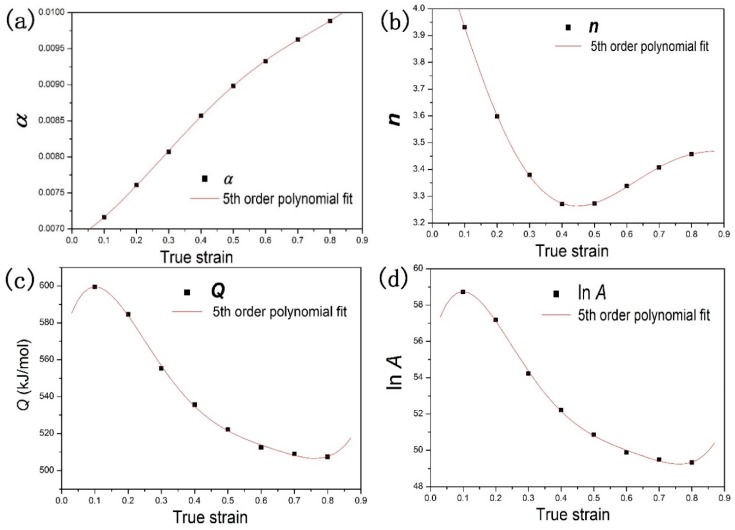
The variation with strain of the material constants: (**a**) *α*, (**b**) *n*, (**c**) *Q*, and (**d**) ln*A*.

Temperature and strain rate were the parameters of concern for elevated temperature stress-strain curves. Strain cannot significantly influence flow stress. However, strain can affect deformation activation energy *Q*. This common phenomenon was studied in Ti-6Al-4V titanium alloy [15], Ti-modified austenitic stainless steel [31], aluminum alloys [14,20,21], magnesium alloy [22,32], and steels [17,18,19]. In isothermal compression tests at elevated temperatures, strain generally influenced flow stress, which was particularly significant at high deformation temperature for TA15 alloy, as can be seen from Figure 8. Therefore, the strain effects cannot be neglected. The results show that strain significantly influenced the materials constants *α*, *β*, *n*, ln*A*, and *Q* in the range of strain.

The strain compensation was considered so that the flow stress of TA15 alloy could be accurately predicted. The model between strain and material constants was established using the polynomial function. The fitted polynomial ranged from two to eight. The optimal fitting model of fifth-order polynomial was selected. The material constants were evaluated at strains from 0.1 to 0.8. Equation (14) is the fifth-order polynomial fitting equation. Generalization and representation would be lost due to the overfitting of higher-order (more than five) polynomials. Table 1 shows the polynomial fitting of the material constants *α*, *n*, *Q*, and ln*A* of TA15 alloy.

(14) $$\alpha =C_{0}+C_{1}\varepsilon +C_{2}\varepsilon^{2}+C_{3}\varepsilon^{3}+C_{4}\varepsilon^{4}+C_{5}\varepsilon^{5}\;n=D_{0}+D_{1}\varepsilon +D_{2}\varepsilon^{2}+D_{3}\varepsilon^{3}+D_{4}\varepsilon^{4}+D_{5}\varepsilon^{5}\;Q=E_{0}+E_{1}\varepsilon +E_{2}\varepsilon^{2}+E_{3}\varepsilon^{3}+E_{4}\varepsilon^{4}+E_{5}\varepsilon^{5}\;\ln A=F_{0}+F_{1}\varepsilon +F_{2}\varepsilon^{2}+F_{3}\varepsilon^{3}+F_{4}\varepsilon^{4}+F_{5}\varepsilon^{5}$$

The constants of materials were calculated by predicting the flow stress through hyperbolic sine function. The constitutive equation of the Zener-Holloman parameter and flow stress was written as follows considering Equations (1) and (10):

(15) $$\sigma =\frac{1}{\alpha }\ln\left\{{\left(\frac{Z}{A}\right)}^{1/n}+{\left[{\left(\frac{Z}{A}\right)}^{2/n}+1\right]}^{1/2}\right\}$$

## 4. Validation of Constitutive Modeling

### 4.1. Results of Constitutive Modeling

**Figure 9:**
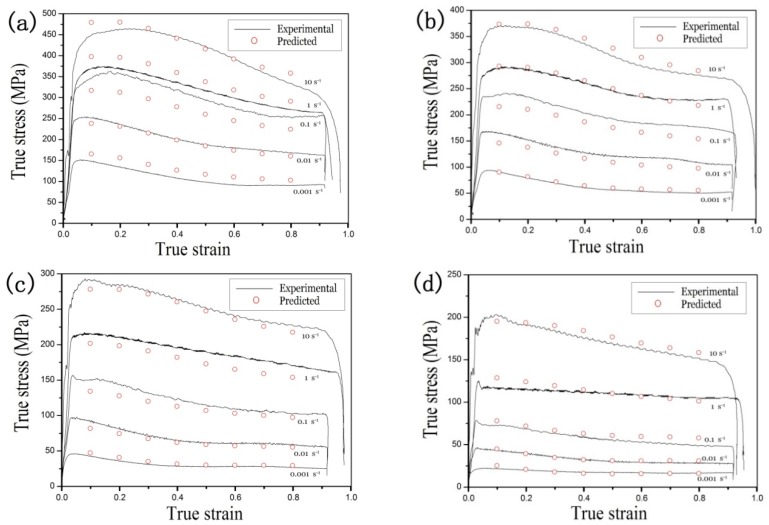
Comparison between the experimental and predicted flow stress at the temperature: (**a**) 1073 K, (**b**) 1123 K, (**c**) 1173 K, and (**d**) 1223 K.

As shown in Figure 9, the experimental data and predicted data were compared to verify the constitutive modeling result considering the strain compensation. The experimental data were consistent with the predicted data in most deformation conditions. In the processing condition (1073 K, when 0.1 s^−1^; Figure 9a), there was variation between the predicted data and the experimental data due to the high nonlinearity of the flow behavior at high temperatures, which was affected by many factors, limiting the accuracy of the applicable range and the flow stress. The model fitting of the materials constants and some deviations may introduce variation into the modeling.

### 4.2. Constitutive Modeling Verification

**Figure 10:**
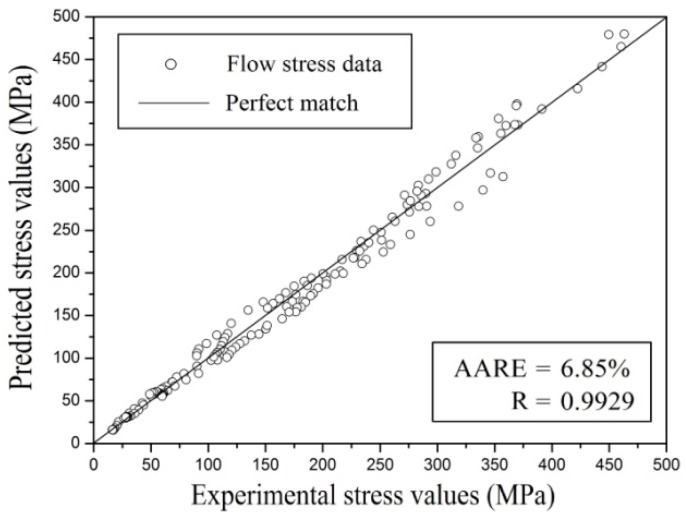
The correlation between predicted data and experimental data over the entire range of strain, strain rate, and temperature.

Figure 10 depicts the correlation of experimental data of flow stress and the predicted data in the constitutive equation incorporating the effect of strain over the entire strain range, strain rate, and temperature. Standard statistical parameters, like *R* and *AARE*, can quantify the predictability of the constitutive equation:

(16) $$R=\frac{\sum_{i=1}^{N}\left(E_{i}-\bar{E}\right)\left(P_{i}-\bar{P}\right)}{\sqrt{\sum_{i=1}^{N}{\left(E_{i}-\bar{E}\right)}^{2}\sum_{i=1}^{N}{\left(P_{i}-\bar{P}\right)}^{2}}}$$

(17) $$AARE=\frac{1}{N}\sum_{i=1}^{N}\left|\frac{E_{i}-P_{i}}{E_{i}}\right|\times 100$$

where *N* is the data number used in the research, $\bar{P}$ and $\bar{E}$ are average values of *P* and *E*, *P* is flow stress predicted from the constitutive equation involving the strain compensation, and *E* is the measured flow stress.

As a common statistical parameter, *R* provides strength information about the relationship between the calculated value and the observed value [21]. Since there is a tendency for the equation to deviate toward lower or higher values, a higher *R* value does not represent better performance. *AARE* was also computed by comparing the relative error. As a result, it is an unbiased statistical parameter used to determine predictability. The values of *AARE* and *R* are 6.85% and 0.9929, respectively, shown in Figure 10. This indicates that the proposed deformation constitutive equation involving strain compensation can estimate flow stress for TA15 titanium alloy accurately.

### 4.3. Prediction of Constitutive Model

Flow stress is complex and associated with complicated metallurgical phenomena, across which hot deformation changes considerably. However, the developed model predicted flow stress in a wide range of strains, strain rates, and temperatures. This can be considered as the major potential of the developed model compared to the analytical, phenomenological, and traditional empirical model that is applied in a specific processing domain [33]. Validating the accuracy of the prediction is good not only for the experimental data but also for the extensional conditions. Because of that higher temperature values can introduce the *β* phase transition in the material, the temperature selected for the specimens prediction accuracy is lower, at 1023 K. The phase change in the microstructure easily produces different stress-strain results, so lower temperatures ensure that the microstructure of the materials is filled with a single *α* phase. A similar study found that the simulation error of extreme points can be decreased by increasing the temperature and strain rate ranges for the near-α titanium alloy [34].

**Figure 11:**
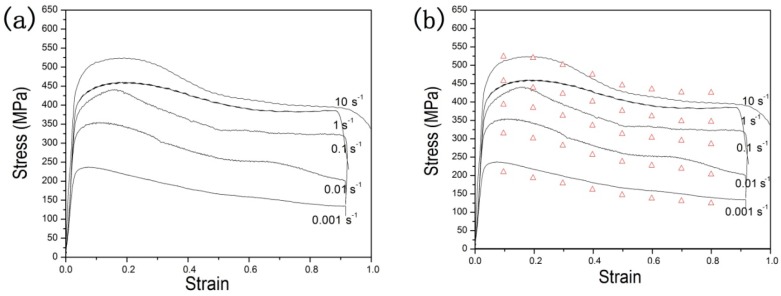
The comparison between the prediction and experimental results at temperature of 1023 K: (**a**) experimental results and (**b**) prediction results of different strain rates.

**Figure 12:**
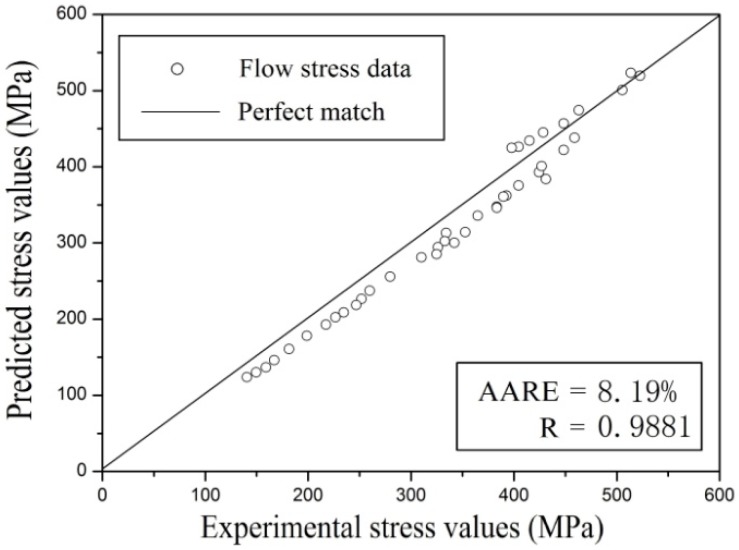
The correlation between predicted and experimental results at temperature of 1023 K.

Therefore, to predict the constitutive model of TA15 titanium alloy, the constitutive model is further extended to predict flow stress at a temperature of 1023 K, which is not included in the previous compression tests. Experimental and predicted flow stresses at a temperature of 1023 K were compared, and results are shown in Figure 11. The same change trend in the stress-strain value is found in the experimental result, and prediction is good at a temperature of 1023 K, as shown in Figure 11b. Similarly, the quantization method with *AARE* and *R*, as mentioned above, was used to evaluate the prediction accuracy. The values of *AARE* and *R* are 8.19% and 0.9881, respectively, shown in Figure 12. This indicates that the proposed constitutive equation considering strain effect has good prediction accuracy for the extended data range for TA15 titanium alloy.

## 5. Conclusions

In this investigation, flow stress at various strain rates and deformation temperatures was predicted by establishing a strain-dependent constitutive equation model for TA15 titanium alloy. The following conclusions were drawn:

(1) TA15 titanium alloy stress was sensitive to deformation temperature and strain rate; the value increased with increasing strain rate and decreased with increased deformation temperature. The experimental result curves exhibited the typical flow behavior, and a constitutive model was introduced based on the Arrhenius equation;
(2) Material constants *α*, *n*, *Q*, and ln*A* were significantly influenced by the strain effect for TA15 titanium alloy, and the relationships between strain and material constants could be described using fifth-order polynomials with a good fitting correlation;
(3) Flow stress at various conditions of deformation was accurately predicted through the constitutive equation incorporating strain compensation. *R* and *AARE* were used to quantify the predictability of the constitutive equation, with values of 6.85% and 0.9929, respectively. The values were 8.19% and 0.9881, respectively, in the prediction using the extended stress-strain data for 1023 K, which proved the high accuracy of the constitutive equation compensated by the strain effect for TA15 titanium alloy.

## Figures and Tables

**Table 1:** Polynomial fitting results of material constants (*α*, *n*, *Q*, and ln*A*).

*α* Coefficient	*n* Coefficient	*Q* Coefficient	ln*A* Coefficient
*C*_0_ = 0.00684	*D*_0_ = 4.29525	*E*_0_ = 567.4279	*F*_0_ = 55.57609
*C*_1_ = 0.00248	*D*_1_ = −3.41358	*E*_1_ = 742.9928	*F*_1_ = 74.06573
*C*_2_ = 0.00883	*D*_2_ = −4.96388	*E*_2_ = −5372.16	*F*_2_ = −541.173
*C*_3_ = −0.01072	*D*_3_ = 29.91754	*E*_3_ = 12837.02	*F*_3_ = 1300.602
*C*_4_ = −0.00216	*D*_4_ = −35.6602	*E*_4_ = −13555.2	*F*_4_ = −1381.13
*C*_5_ = 0.00544	*D*_5_ = 13.30015	*E*_5_ = 5382.073	*F*_5_ = 551.388
